# Supplementary material for: All-at-once RNA folding with 3D motif prediction framed by evolutionary information
Source: Nat Methods. 2025 Oct 3;22(10):2094–106. doi: 10.1038/s41592-025-02833-w (PMC12510878; doi:10.1038/s41592-025-02833-w)
Supplement: Supplementary file 3 — Source code to run CaCoFold-R3D. Data and results. [file 41592_2025_2833_MOESM3_ESM.gz › supplemental_material/results/CaCoFold-R3D/CaCoFold-R3D_Rfam/RF02285_TtnuCD8.R2R.sto.pdf]

RF02285_TtnuCD8

5′ ● ● A A Y A U ● C C A G U U C U G C U G C U G A A A A C A G C U U G A A G A ○ A A Y U U U R C U U A C C ● U U C U G A
